# On neotypes and nomina nova: commentary on “Comparative analysis of *Faecalibacterium prausnitzii* genomes shows a high level of genome plasticity and warrants separation into new species-level taxa”, by C.B. Fitzgerald et al. (BMC Genomics (2018) 19:931)

**DOI:** 10.1186/s12864-020-6680-3

**Published:** 2020-04-30

**Authors:** Aharon Oren, George M. Garrity

**Affiliations:** 1 The Institute of Life Sciences, The Hebrew University of Jerusalem, The Edmond J. Safra Campus, 9190401 Jerusalem, Israel; 2 Department of Microbiology & Molecular Genetics, Biomedical Physical Sciences, Michigan State University, East Lansing, MI 48824-4320 USA

**Keywords:** *Faecalibacterium prausnitzii*, *Faecalibacterium moorei*, Neotype strains, Nomina nova, International Code of Nomenclature of Prokaryotes

## Abstract

In their paper entitled “Comparative analysis of *Faecalibacterium prausnitzii* genomes shows a high level of genome plasticity and warrants separation into new species-level taxa” (BMC Genomics (2018) 19:931), Fitzgerald et al. proposed a neotype strain for *F. prausnitzii* (strain A2–165 = DSM 17677 = JCM 31915) and assigned strain ATCC 27768 = NCIMB 13872 to a newly established taxon, *Faecalibacterium moorei* nom. Nov. These proposals contravene the Rules of the International Code of Nomenclature of Prokaryotes (ICNP). Neotype strains can only be established following a formal proposal in the International Journal of Systematic and Evolutionary Microbiology in accordance with Rule 18c and Appendix 7 of the ICNP. A proposed neotype becomes an established neotype after 2 years, provided that no objections were submitted to the Judicial Commisson of the International Committee on Systematics of Prokaryotes within the first year following publication of the request. *F. moorei* as proposed by Fitzgerald et al. is a later homotypic synonym of *F. prausnitzii*. It cannot be a ‘nom. nov.’ (nomen novum): based on Rule 34a of the ICNP: this term is only used when an author transfers a species to another genus or a subspecies to another species as a new combination, but the original specific epithet cannot be used as ‘comb. nov.’ (combinatio nova) as a result of homonymy. Moreover, ATCC 27768 and NCIMB 13872 cannot be proposed as the type strain of *F. moorei* as these remain permanently associated with the type strain of *F. prausnitzii* unless the Judicial Commission of the ICSP will decide otherwise.

## Main text

In their paper entitled “Comparative analysis of *Faecalibacterium prausnitzii* genomes shows a high level of genome plasticity and warrants separation into new species-level taxa” [1], Fitzgerald et al. performed a comparative study of multiple isolates of the important human gut bacterium *Faecalibacterium prausnitzii*. They identified two phylogroups. Phylogroup II is the best-known and most extensively studied group. The type strain of *F. prausnitzii* Duncan et al. 2002 (ATCC 27768^T^ = NCIMB 13872^T^) [2] belongs to the phylogroup I. The authors then proposed a neotype strain for *F. prausnitzii* (strain A2–165 = DSM 17677 = JCM 31915) and provided an emended description of the species, while assigning strain ATCC 27768 = NCIMB 13872 to a newly established taxon, *Faecalibacterium moorei* nom. nov.

In these proposals, Fitzgerald et al. use the terminology and the currently accepted way to formulate proposals for the establishment of new names of prokaryotic taxa as defined in the International Code of Nomenclature of Prokaryotes (ICNP) [3], as approved by the International Committee on Systematics of Prokaryotes (the ICSP). Unfortunately, the authors apparently did not familiarize themselves with the meaning of the terms ‘neotype strain’ and ‘nom. nov.’ (nomen novum) as defined by the Rules of the ICNP. As a result, these terms were misused and the proposals effectively published by the authors in BMC Genomics cannot be accepted for valid publication in the *International Journal of Systematic and Evolutionary Microbiology* (IJSEM).

If a strain on which the original description was based cannot be found, a neotype strain may be proposed in the IJSEM, together with citation of the author(s) of the name, a description or reference to an effectively published description, and a record of the permanently established culture collection(s) where the strain is deposited. The author should show that a careful search for the strains used in the original description has been made and that none of them can be found. The author should also demonstrate that the proposed neotype agrees closely with the description given by the original author. The neotype becomes established (established neotype) 2 years after the date of its publication in the IJSEM, provided that there are no objections, which must be referred within the first year of the publication of the neotype to the Judicial Commission of the ICSP for consideration (Rule 18c). A strain suggested as a neotype but not formally proposed in accordance with the requirements has no standing in nomenclature until formally proposed and established (Rule 18d). Appendix 5 of the ICNP lists 24 established neotypes discussed by the Judicial Commission between the years 1958 and 1999. Neotypes for three more species (*Eubacterium rectale*, *Myxococcus macrosporus* and *Myxococcus stipitatus*), proposed in 2008 and 2009 were established without objections. In the case of *Faecalibacterium prausnitzii* the strain on which the original description was based is readily available. Indeed, some neotype strains were established for other reasons in the opinions issued by the Judicial Commission. Therefore, Fitzgerald et al. could have submitted a Request for an Opinion to the Judicial Commission if in their opinion, the current type strain of the species is not appropriate, but they failed to do so. It must also be remembered that the nomenclatural type is that element of the taxon with which the name is permanently associated, and is not necessarily the most typical or representative element of the taxon (Rule 15 of the ICNP).

For the formal description of the new species-level taxon *Faecalibacterium moorei*, Fitzgerald et al. chose the designation ‘nom. nov.’ instead of the correct ‘sp. nov.’. The designation ‘nom. nov. (= nomen novum), based on Rule 34a of the ICNP, is only used when an author transfers a species to another genus or a subspecies to another species as a new combination, but the original specific epithet cannot be used as ‘comb. nov.’ (combinatio nova) as a result of homonymy. Then the author is obliged to substitute a new specific epithet, and the abbreviation ‘nom. nov.’ (nomen novum) is used instead. Establishment of nomina nova is a rare event. The last cases recorded include the proposal of *Chryseobacterium ginsengiterrae* (Hoang et al. 2015) Hahnke et al. 2016 (basonym: *Epilithonimonas ginsengisoli* Hoang et al. 2015) and *Chryseobacterium halperniae* (Shakéd et al. 2010) Hahnke et al. 2016 (basonym: *Epilithonimonas lactis* Shakéd et al. 2010) [4, 5]. Another relatively recent one is *Cronobacter zurichensis* (Stephan et al. 2007) Brady et al. 2013 (basonym: *Enterobacter turicensis* Stephan et al. 2007).

Journals such as BMC Genomics are suitable platforms for the effective publications of names of new taxa of prokaryotes in accordance with Rule 25a of the ICSP. If the proposals are prepared in accordance with the Rules of the Code, notably rules 27 and 30, the names can be submitted for validation in the IJSEM. It is the task of the authors, assisted by the editors and reviewers of the journal, to take care that the proposals are correctly formulated. The List Editors of the IJSEM have limited authority to make corrections in the Validation Lists. It is unfortunate that proposals made by Fitzgerald et al. were incorrectly formulated and contravened the Rules of the ICNP, and this was probably not spotted by the reviewers and by the editor who handled the submission. It is especially serious that the paper creates the false impression that strain ATCC 27768 = NCIMB 13872 is no longer the type strain of *Faecalibacterium prausnitzii*, and that a neotype strain was established, an act that can only be performed by a formal proposal in the IJSEM in accordance with Rule 18c and Appendix 7 of the ICNP. As noted above, a proposed neotype becomes an established neotype after 2 years, provided that no objections were submitted to the Judicial Commisson within the first year following publication of the request. No formal request to establish a neotype strain was yet published by Fitzgerald and coworkers; therefore their emended description of *Faecalibacterium prausnitzii* contains severe mistakes. The effective publication of *Faecalibacterium moorei* as a new species cannot be approved for valid publication of the name as it is a later homotypic synonym of *Faecalibacterium prausnitzii*. Moreover, the designation ‘nom. nov.’ is not appropriate here. The authors may propose a new species as follows: Description of *Faecalibacterium moorei* sp. nov. (moo’re.i. N.L. gen. *n. moorei* of Moore …), to be followed by the description (‘protologue’) of the new taxon and designation of the type strain with culture collection deposition numbers; however, that cannot be ATCC 27768 and NCIMB 13872 as these remain permanently associated with the type strain of *Faecalibacterium prausnitzii* unless the Judicial Commission of the ICSP will decide otherwise.

## Data Availability

Not applicable.

## Abbreviations

ATCC: American Type Culture Collection
DSM: Deutsche Sammlung von Miroorganismen
NCIMB: National Collection of Industrial Food and Marine Bacteria
JCM: Japan Collection of Microorganisms
ICNP: International Code of Nomenclature of Prokaryotes
ICSP: International Committee on Systematics of Prokaryotes
IJSEM: International Journal of Systematic and Evolutionary Microbiology
