# Supplementary material for: On neotypes and nomina nova: commentary on “Comparative analysis of Faecalibacterium prausnitzii genomes shows a high level of genome plasticity and warrants separation into new species-level taxa”, by C.B. Fitzgerald et al. (BMC Genomics (2018) 19:931)
Source: BMC Genomics. 2020 Apr 30;21:335. doi: 10.1186/s12864-020-6680-3 (PMC7193374; doi:10.1186/s12864-020-6680-3)
Supplement: Related texts

# Reply: Response to: On neotypes and nomina nova: commentary on “Comparative analysis of Faecalibacterium prausnitzii genomes shows a high level of genome plasticity and warrants separation into new species-level taxa”, by C.B. Fitzgerald et al. (BMC Genomics (2018) 19:931)

**Authors:** Colin Hill, Cormac Brian Fitzgerald, Andrey N. Shkoporov

We welcome this discussion of our article. The authors of the commentary are experts in this field and we are happy to abide by their advice. The primary reason that we proposed that *Faecalibacterium prausnitzii* A2–165 should remain as the representative strain for F. prausnitzii rather than be re-designated as *Faecalibacterium moorei* was because of the number of papers that have already been published on this strain under the *F. prausnitzii* nomenclature. We believed that reclassifying the phylogroup which contains the current type strain, ATCC 27766, would cause less confusion in the future and allow for a more consistent link between previous and future studies involving *Faecalibacterium*.

We fully recognise that it is important to abide by the current system and rules for reclassifying prokaryotes and that the use of the words ‘neotypes’ and ‘nomina nova’ in this instance were incorrect.
